# Knowledge, attitude, practice and associated factors of health professionals towards podoconiosis in Gamo zone, Ethiopia, 2019

**DOI:** 10.1186/s13047-021-00464-w

**Published:** 2021-04-14

**Authors:** Chuchu Churko, Mekuria Asnakew Asfaw, Abayneh Tunje, Eyayou Girma, Zerihun Zerdo

**Affiliations:** 1 Collaborative Research and Training Center for Neglected Tropical Diseases, College of Medicine and Health Sciences, Arba Minch University, P.O. Box 21, Arba Minch, Ethiopia; 2 School of public health, College of Medicine and Health Sciences, Arba Minch University, P.O. Box 21, Arba Minch, Ethiopia; 3 Department of Biomedical Sciences, College of Medicine and Health Sciences, Arba Minch University, P.O. Box 21, Arba Minch, Ethiopia; 4 Department of Medical Laboratory Technology, College of Medicine and Health Sciences, Arba Minch University, P.O. Box 21, Arba Minch, Ethiopia

**Keywords:** Podoconiosis, Health professionals, Ethiopia

## Abstract

**Background:**

Podoconiosis is entirely preventable, non-communicable disease with high potential of elimination. The prevalence of podoconiosis in Ethiopia was 7.45%. One of the pillars for elimination of podoconiosis is morbidity control and management. Therefore, the present study aimed to assess the knowledge, attitude, practices and associated factors of health professionals towards podoconiosis cause, prevention and treatments.

**Methods:**

Facility based cross-sectional study was conducted. The source population was all health professionals currently working in public health facilities. The final estimated sample size was 349. A pretested self-administrated structured questionnaire was used to collect the data. The data were coded, entered, and cleaned by using Epi.info version7, and analyzed by using SPSS version 20.

**Result:**

A total of 320 health professionals participated in the study. Sixty eight (23.1%) health professionals had poor knowledge towards podoconiosis. Seventy (21.9%) identified podoconiosis as infectious disease. Profession, address of health facility, service year and attitude of participants were significantly associated with knowledge towards podoconiosis. More than half (56%) had favorable attitude towards podoconiosis patients. Knowledge score (95%CI: 1.389, 4.059, *p*-value = 0.002) was the independent predictor for attitude status. Very few (11.6%) respondents treated podoconiosis patients. Age group 45 years old and above and training on lymphedema morbidity management and disability prevention were significantly associated with clinical experience in treating affected patients, (AOR = 17.345; 95%CI: 4.62, 65.119) and (AOR = 7.385; 95%CI: 2.5, 21.797), respectively.

**Conclusion:**

Despite, high percent of good knowledge of health professionals towards podoconiosis, clinical experience of health professionals in treating podoconiosis patients was very low. In-service trainings will be given for health professionals to improve treatment. In podoconiosis endemic districts hygiene supplies and other referencing materials should be made available for podoconiosis case management.

## Background

Neglected tropical diseases (NTD) affect more than 1 billion people globally and World Health Organization (WHO) African Region accounts about half of this global burden. Podoconiosis is greatly predominant in tropical and subtropical regions and affect mainly the low income and impoverished communities with average prevalence of 5–10% of world population living on irritant soil as per WHO estimates. Neglected tropical diseases are identified by their slowly evolving symptoms that often lead to devastating complications. By impairing the physical and intellectual capacities of the affected persons, these diseases perpetuate the cycle of poverty and negatively affect socioeconomic development [1].

Podoconiosis, endemic non-filarial elephantiasis, is one of the neglected tropical diseases which is caused by prolonged exposure of barefoot to red clay soil of volcanic origin [2]. It affects the poorest community in tropical and subtropical areas in the world [3], but it is easily preventable and treatable by using simple practical measures like regular and consistent wearing of proper shoes and daily foot washing with soap and water [3].

The disease affects men and women equally in most poorest societies in tropical and sub-tropical areas [4]. Most of the community based studies have shown that the onset of clinical signs and symptoms happen in the first and second decades and gradually increases in prevalence up to the sixth decade [4]. People who do not wear shoes regularly due to poverty or other cultural reasons are at higher risk for podoconiosis [4].

Podoconiosis causes bilateral yet asymmetrical leg swelling with firm nodules. Early onset symptoms may include itching, tingling, widening of the forefoot, and swelling which then advance to soft edema, skin fibrosis, papillomatosis, and nodule formation resembling moss, giving rise to the disease’s alternate name of “mossy foot” in some regions of the world [5]. As with other form of lymphedema, chronic disease can lead to rigid toes, ulceration of lower leg, and bacterial super infection. During acute episodes of adenolymphangitis, patients may develop fevers, extremity warmth, redness, and pain. These episodes are extremely debilitating and account for many days of activity and economic productivity loss each year [5].

Endemic non-filarial elephantiasis or podoconiosis has severe social, economic and health consequences. According to the study conducted in Ethiopia, Wolayta zone revealed that the annual economic cost of the disease in an area with 1.7 million people was more than 16 million US dollars. When extrapolated to the national population, this finding reflects a corresponding cost of more than 200 million US dollars. People affected with podoconiosis were found to lose 45% of their economically productive time because of illness associated with the disease. Majority of people living with podoconiosis in Ethiopia experience an episode of acute inflammation, which may be triggered by bacterial, viral or fungal infection, at least once per year. Such types of acute attacks are characterized by hot, painful and reddened swelling. Since podoconiosis patients become bedridden during such attacks, it leads to loss of economic productivity [6].

Recently a systemic review on global epidemiology on podoconiosis conducted by Kebede Derib and his colleagues reported that the overall podoconiosis prevalence ranges from 0.10 to 8.08% globally. The highest prevalence was in African region with substantially higher in adults than in children and adolescents. Their report showed that the prevalence of podoconiosis in Cameroon is 8.08, 7.45% in Ethiopia, 4.52% in Uganda, 3.87% in Kenya and 2.51% in Tanzania [7].

According to WHO report stigma from podoconiosis is pronounced; patients being excluded from school, local meetings, churches and mosques, and barred from marriage with unaffected individuals [8]. Although podoconiosis has severe social stigma, physical disability and economic unproductivity, the disease is neglected by global community. Most studies show that podoconiosis is a common public health problem in African regions which affects mostly poorest community of the tropics with the highest prevalence. Despite the high prevalence and terrible impact of podoconiosis, it is not recognized by most governmental and nongovernmental organizations [9] and there are few control and prevention initiatives in Ethiopia. These initiatives are limited in certain parts of Ethiopia. In Wolayta zone, the Mossy Foot Treatment and Prevention Association (MFTPA) has been documented as effective continuing podoconiosis community program model since 1998 as compared to the WHO Innovative Care for Chronic Conditions (ICCC) Framework [10]. The MFTPA program involves prevention (distribution of shoes to children, adult shoemaking), treatment (shoe wearing education integrated into clinics/hygiene), and rehabilitation (microcredit, training) activities at the community-level across one zone. In June 2010, the first podoconiosis program in Northern Ethiopia was started in Debre Markos, East Gojam Zone in an effort to take the experiences of MFTPA and develop a program specific to the context of Northern Ethiopia. The program in Debre Markos aims to address podoconiosis prevention, awareness, and care and support activities [11].

Ethiopia also developed a guideline for lymphatic filariasis and podoconiosis morbidity management and disability prevention that contribute its part in the control and elimination of podoconiosis. The guideline is helpful for general medical practitioners, health officers, nurses and other mid-level health practitioners in their efforts to reduce morbidity and disability of podoconiosis [12].

Despite the above mentioned efforts made by government of Ethiopia, there was no scientific data that show level of knowledge, attitude and practices of health professionals towards podoconiosis in the study area. Therefore the aim of this study was to assess the knowledge, attitude, practices and associated factors of health professionals towards podoconiosis in Gamo zone, Southern Ethiopia.

## Methods and materials

### Study setting and period

The study was conducted in Gamo zone, Southern Ethiopia from September 1 to December 30, 2019. Gamo zone is one of the zones in the Southern Nations, Nationalities, and Peoples' Region of Ethiopia. According to the 2007 Ethiopian central statistics agency Census, the zone had a total population of 1,341,901 of whom 668,230 were males and 673,671 were females. Majority of the population 1,292,653 (96.33%) live in rural area.

The current profile indicated that there are 5 hospitals, 33 private clinics and 53 health centers in Gamo zone. There are a total of 3767 and 587 health professionals and health extension workers in the zone, respectively.

### Study design

- Facility based cross-sectional design was employed

### Study population

All health professionals in randomly selected districts were our study population.

### Eligibility criteria

#### Inclusion criteria

All health professionals working currently in public health facilities in randomly selected districts were included in the study.

#### Exclusion criteria

- Anesthesia and environmental health professionals.
- Eligible health professionals not willing to participate and absent during study period.

### Sample size determination

Sample size was determined by using single population proportion formula with the assumption presented below:

$$ \mathrm{N}=\frac{{{\mathrm{z}}^2}_{\upalpha /2}\ast \mathrm{P}\left[1-\mathrm{P}\right]}{{\mathrm{d}}^2} $$

Where,

N = initial sample size

Z_α/2_ = significance level at 95% confidence interval = 1.96

*P* = 50% was used to get the highest sample size.

Degree of margin = 5%

$$ \mathrm{N}=\frac{1.{96}^2\ast 0.5\ \left[1-0.5\right]}{0.05^2}=\frac{3.8416\ast 0.25}{0.0025}=348 $$

Since the total population was less than 10,000, we used finite population correction formula.

As n = n0 *N, 384*3767 = 349, the final estimated sample size was 349.

$$ \mathrm{n}0+\left(\mathrm{N}-1\right)\ 384+\left(3767-1\right) $$

### Sampling procedure

**Fig. 1:**
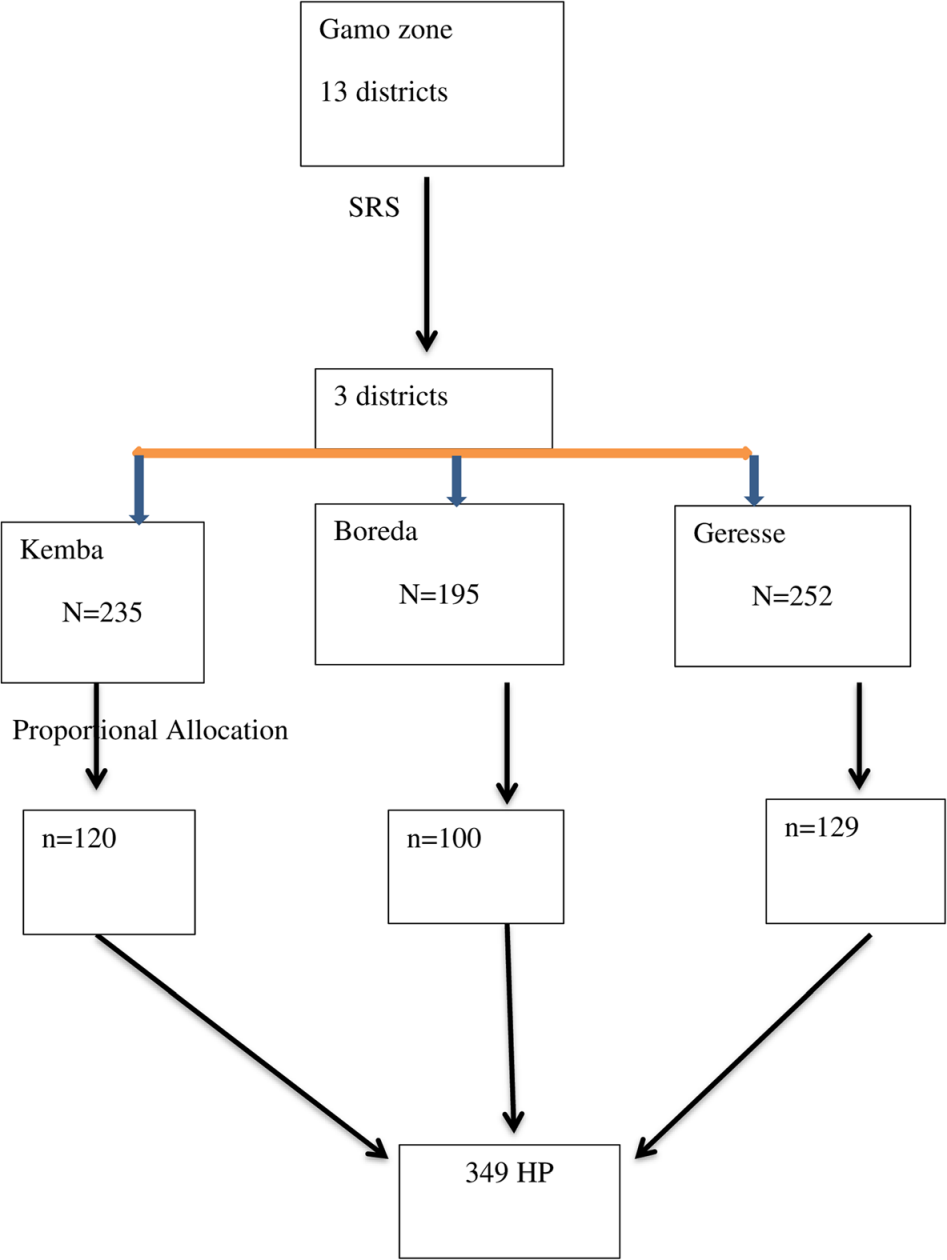
schematic presentation of sampling procedure of knowledge, attitude, practice and associated factors of health professionals towards podoconiosis prevention and control, 2019. Key: N = number of health professionals in the districts, n = sample size required from each district

From total of thirteen districts in Gamo zone, Southern Ethiopia, three districts (Boreda, Kamba and Geresse) were randomly selected for the current survey. Then total number of health professionals who were working in public health institutions in each selected districts were identified. We proportionally allocated the sample size to these districts. Finally, respondents’ selection was made by using lottery method (Fig. 1).

### Study variables

#### Dependent variables

Knowledge (good/poor)

Attitude (favorable or unfavorable)

Practice (ever treated podoconiosis yes or no)

#### Independent variables

Socio-demographic variables**:** age, sex, marital status, type of health facility, level of health facility, type of profession, service year of health professionals (HP), availability of drugs and supplies/treatment guidelines on podoconiosis, training on podoconiosis.

### Operational definitions

**Knowledge** was assessed in terms of what a person knows about podoconiosis and whether this knowledge was true or false. A person had good knowledge if he/she scored 75% or more of the knowledge question and poor knowledge if he/she answered less than 75% of the knowledge questions.

**Attitude** questions was measured by using likart scale as strongly agree, agree, neutral, disagree and strongly disagree. Those individuals who agree 75% or more of attitude questions was categorized as favorable attitude and participants who agree below the 75% as categorized in to unfavorable attitude.

**Practice:** those health professionals who ever treated podoconiosis (yes or no).

**Health professionals:** refer to people who had formal professional training in health discipline (Lab techni., pharmacy tech., health officers, nurses and physicians), and have been accredited and given legal permit to practice either in public or private facilities.

### Data collection

Data were collected by using self-administered structured questionnaire. The questionnaire covered four sections, socio-demographic characteristics, knowledge about podoconiosis cause, prevention and treatment, attitude towards podoconiosis patients and experiences in treating podoconiosis patients. Ten data collectors and three supervisors who had BSc and above educational background, experience on data collection and working in academic environment were recruited for data collection.

### Data quality control

To maintain data quality, data collection tool was used. The questionnaire was prepared in English. Pretesting was done on 5% of the total sample size. Four day training was given for the data collectors and the supervisors. Daily supervision was carried out to check the completeness and consistency of the questionnaire.

### Data processing and analysis

Epi info version 7 was used for coding, entering and cleaning the collected data and the data was imported to and analyzed by using SPSS version 20. Bivariate analysis was done to determine the associations between each independent variables and outcome variables. All associated factors with *p*-value less than 0.25 during bivariate analysis were entered in to multivariable logistic regression model. Odds ratio with 95% confidence intervals was used to see the strength of association between different variables. *P*-value and 95% confidence interval (CI) for odds ratio (OR) was used in deciding the significance of the associations. Before inclusion of independent factors, multi-collinearity was checked using cutoff points Variance Inflation Factor (VIF) < 10 and normality was checked by Q-Q probability plots. Hosmer-Lemeshow goodness of fit was also checked for the model at *P*-value > 0.05. Finally data were presented in text, tables and graphs.

### Ethical approval

Ethical approval for this study was obtained from institute research board (IRB), Medicine and Health Science College, Arba Minch University. Support letter was obtained from Gamo zone health department and the district health offices to facilitate the data collection. Informed verbal consent was obtained from each study participants before proceeding to data collection.

## Result

### Socio-demographic characteristics of the respondents

Of the total 349 estimated sample, 320 health professionals filled the questionnaire with a response rate of 92%. The recent 8% of eligible participants did not fill the questionnaire because of different reasons like annual leave, sickness and went outreach programs. Majority 202 (63.1%) of the study subjects were in the age group 25 to 34 years old. From the total participants, 205 (64.1%) were males. Regarding professional background, about half 162 (50.6%) of the respondents were diploma nurses (Table 1).

**Table 1:** Socio-demographic characteristics of health professionals in Gamo zone, Southern Ethiopia, 2019

Variables	Categories	Frequency	Percentage
Age	25–34	202	63.1%
	35–44	106	33.1%
	45 and above	12	3.8%
	Total	320	100%
Sex	Female	115	35.9%
	Male	205	64.1%
Profession	BSc nurse	58	18.1%
	HO^a^	57	17.8%
	Diploma nurse	162	50.6%
	Pharmacy	9	2.8%
	Lab technician	18	5.7%
	Midwives	16	5%
Marital status	Single	116	36.3%
	Married	200	62.5%
	Others^b^	4	1.2%
length of service	1 to 5 years	171	53.4%
	6 to 10 years	98	30.6%
	11 and above years	51	16%

^a^Health Officer, ^b^Divorced and widowed

### Knowledge of health professionals towards podoconiosis prevention and control

All (100%) of the respondents had heard about podoconiosis. Seventy (21.9%) health professionals responded that podoconiosis is infectious disease, which is not correct. Thirty eight (11.9%) and 71 (22.1%) said that the disease is caused by parasite and curse/evil eye, respectively, which is also not correct. Forty five percent of participants incorrectly answered about risk factors for podoconiosis. In general, 68 (23.1%) health professionals had poor knowledge towards prevention and control of podoconiosis (Table 2).

**Table 2:** knowledge of health professionals on podoconiosis prevention and control in Gamo zone, Southern Ethiopia, 2019. (*N* = 320)

Variables	Categories	Frequency (n)	Percentage (%)
Podoconiosis is infectious disease	Yes	70	21.9
	No	250	78.1
Cause of podoconiosis	Hereditary disease	57	17.8
	Parasitic disease	71	22.1
	Curse/evil eye	38	11.9
	Soil particle	154	48.1
Risk factor for podoconiosis (multiple options)	Not wearing shoes	176	55
	Improper foot hygiene	188	58.8
	Not using bed net	144	45
Signs and symptoms (multiple options)	Lower leg swelling	314	98.1
	Upper arm swelling	193	60.3
	Scrotal swelling	40	12.5
	Breast swelling	71	22.2
Prevention measures of podoconiosis (multiple options)	Avoiding stepping on dead animals	10	3.1
	Proper and regular shoe wearing	193	60.3
	Avoiding prolonged bare foot contact with irritant red clay soil	200	62.5
	Daily foot washing	268	83.8
	Covering floors	74	23.1
	Use bed net	81	25.3
Management of podoconiosis patients (multiple options)	Foot hygiene	124	38.8
	Elevation and exercise	192	60
	Bandaging	133	41.6
	Skin care	153	47.8
	Wound care	168	52.5
	counseling	198	61.9
Knowledge status	Poor	74	23.1
	Good	246	76.9

### Attitude of health professionals on podoconiosis patients

**Fig. 2:**
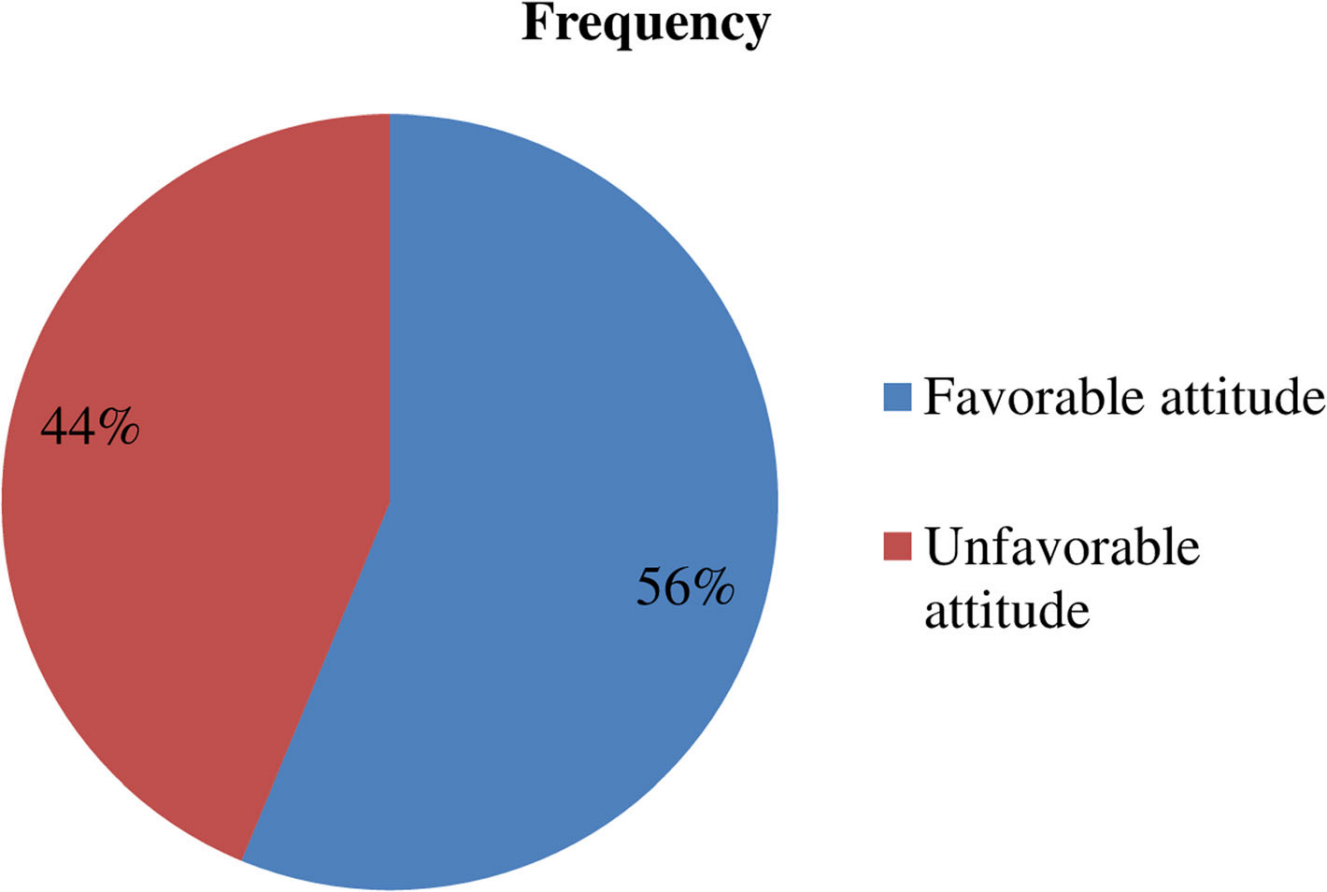
attitude of respondents towards podoconiosis patients in Gamo zone Southern Ethiopia, 2019

More than half 180/320 (56%) of study subjects had favorable attitude towards podoconiosis patients. Most of health professionals 191/320 (59.7%) disagreed that they had adequate knowledge and skill to give care and treatment for podoconiosis patients. Forty seven (14.7%) believed they may acquire podoconiosis from a patient and 32 (10%) of the study participants were neutral to whether they acquire the disease or not (Table 3 and Fig. 2).

**Table 3:** Attitude of health professionals on podoconiosis prevention and control measures

Variables	Strongly agree (1)	Agree (2)	Neutral (3)	Disagree (4)	Strongly disagree (5)
I feel I had adequate knowledge and skill to give care and treatment for podoconiosis patients	1(0.3%)	26(8.1%)	28(8.8%)	191(59.7%)	74(23.1%)
I may acquire podoconiosis if I am in contact with podoconiosis patients	0	47(14.7%)	32(10%)	182(56.9%)	59(18.4%)
I feel podoconiosis patients deserve care and support	146(45.6%)	167(52.2%)	7(2.2%)	0	0
People with podoconiosis should be legally separated from others to protect the public health	0	3(0.9%)	46(14.5%)	173(54%)	98(30.6%)
I feel comfort if I buy food or items from a shop keeper with podoconiosis	77(24.1%)	142(44.4%)	57(17.8%)	42(13.1%)	2(0.6%)
People will appreciate me if they knew I treated podoconiosis patients	43(13.4%)	185(57.8%)	72(22.5%)	20(6.3%)	0
The family of the person with podoconiosis should be blamed for passing on the disease	0	8(2.5%)	24(7.5%)	178(55.6%)	110(34.4%)
The family of the person with podoconiosis is cursed	0	6(1.9%)	65(20.3%)	122(38%)	127(39.7%)
The family of the person with podoconiosis should be isolated	0	1(0.3%)	36(11.2%)	159(49.7%)	124(38.8%)
People will isolate my family members if they knew I treated podoconiosis patient	0	0	21(6.6%)	161(50.3%)	138(43.1%)

### Practice of health professionals in the treatment of podoconiosis patients

With respect to practice, very few, 37/320 (11.6%) health professionals had treated podoconiosis patients. Majority, 283/320 (88.4%) did not ever treat podoconiosis patients due to different reasons, 150/283 (53%) no LMMDP (Lymphoedema and morbidity management and disability prevention) materials, 100/283 (35.3%) no training given and 33/283 (11.8%), no case. Most, 311/320 (97.2%) of health professionals had no LMMDP guideline for managing patients in their health facilities (Table 4).

**Table 4:** practice of study subjects towards management of podoconiosis cases in Gamo zone, SNNPR, Ethiopia, 2019

Variables	Category	Frequency	Percent
Ever treated podoconiosis patients	Yes	37	11.6%
	No	283	88.4%
	Total	320	100%
If treated podoconiosis patients, services given (more than one answer possible)	Antibiotic	27	8.4%
	Cleaning	28	8.8%
	Massaging	13	4%
	Bandaging	5	1.6%
If not treated, why? More than one answer possible	Not trained	100	35.3%
	No MMDP material	150	53%
	No case	33	11.7%
Availability MMDP guideline	Yes	9	2.8%
	No	311	97.2%

### Factors associated with knowledge, attitude and practice of health professionals towards podoconiosis

After adjusting for profession, address of health facility and years of service; health professionals whose profession was BSc nurse, health officers (HO), and diploma nurse had significantly higher chance to have good knowledge (AOR = 11.44, 95%CI: 3.79, 34.547), (AOR = 24.977, 95%CI: 6.412, 97.25) and (AOR = 3.522, 95%CI: 1.65, 7.5), respectively (Table 5).

**Table 5:** Multivariable logistic regression analysis of knowledge status of health professionals towards podoconiosis in Gamo zone, 2019

Variables	Category	Knowledge status			COR(95%CI), *p*-value	AOR(95%CI)	*P*-value
		Good	Poor	Total			
Trained on LMMDP	Yes	17	1	18	Reference		0.50
	No	229	73	302	0.185(0.024, 1.41), 0.103	0.484(0.058, 4.032)	
	Total	246	74	320			
Sex	Male	164	41	205	Reference		
	Female	82	33	115	0.621(0.366, 1.055), 0.078	0.803(0.434, 1.485)	0.485
	Total	246	74	320			
Profession	BSc nurse	52	6	58	9.967(3.537, 28.08), 0.001	11.44(3.79, 34.547)	0.001**
	Health officer	54	3	57	20.7(5.597, 76.56), 0.001	24.977(6.412, 97.25)	0.001**
	Diploma nurse	120	42	162	3.286(1.64, 6.58), 0.001	3.522(1.65, 7.5)	0.001**
	Others*	20	23	43	Reference		
	Total	246	74	320			
Service year	1–4	82	47	129	Reference		
	5–9	106	20	126	3.038(1.67, 5.52), 0.001	3.124(1.612, 6.05)	0.001**
	10 and above year	58	7	65	4.749(2.01, 11.249), 0.001	4.4(1.769, 10.951)	0.001**
	Total	246	74	320			
Address of health facility	Rural	175	64	239	Reference		
	Urban	71	10	81	2.597(1.262, 5.341), 0.01	0.938(0.358, 2.45)	0.896
	Total	246	74	320			
Attitude	Unfavorable	95	45	140	Reference		
	Favorable	151	29	180	2.466(1.448, 4.2), 0.001	2.63(1.448, 4.78)	0.002**
	Total			320			

*Others: midwife, laboratory and pharmacy technician, **strongly significant

Regarding attitude, knowledge status was statistically significantly associated with attitude of study subjects. Those individuals who had good knowledge were 2.6 times more likely to have favorable attitude when compared to poor knowledge scorer (95%CI: 1.389, 4.059, *p*-value = 0.002) (Table 6).

**Table 6:** Multivariable logistic analysis for attitude score of study subject in Gamo zone, SNNPR, Ethiopia, 2019

Variable	Category	Attitude			COR(95%CI), *p*-value	AOR(95%CI)	*P*-value
		Favorable	Unfavorable	Total			
Sex	Female	56	59	115	0.62(0.39, 0.98), 0.042	0.659(0.412, 1.054)	0.082
	Male	124	81	205	Reference		
	Total	180	140	320			
Knowledge status	Good	151	95	246	2.466(1.448, 4.201), 0.001	2.374(1.389, 4.059)	0.002**
	Poor	29	45	74		Reference	
	Total	180	140	320			

**strongly significant

In multivariable logistic regression analysis, compared to respondents less than 34 years old, respondents age 45 years old and above were significantly more likely to have treated podoconiosis patients (AOR = 17.345; 95%CI: 4.62, 65.119). Health professionals who trained on LMMDP have 7.4 times chance of treating podoconiosis patients than respondents who did not train on LMMDP (AOR = 7.385; 95%CI: 2.5, 21.797) (Table 7).

**Table 7:** Factors associated with practice of health professionals towards podoconiosis treatment

Variables	Category	Ever treated podo cases			COR(95%CI), *p*-value	AOR (95%CI)	*P*-value
		Yes	No	Total			
Trained on LMMDP	Yes	9	9	18	9.786(3.59, 26.66), 0.001	7.385(2.5, 21.797)	0.001**
	No	28	274	302	Reference		
	Total	37	283	320			
Age category	25–34	13	189	202	Reference		
	35–44	17	89	106	2.77(1.29, 5.967), 0.009	2.115(0.942, 4.75)	0.07
	45 and above	7	5	12	20.35(5.67, 73.048), 0.001	17.345(4.62, 65.119)	0.001**
	Total	37	283	320			
Service year	1–4	8	121	129	Reference		
	5–9	10	116	126	1.3(0.497, 3.42), 0.59	1.065(0.348, 3.257)	0.912
	10 and above	19	46	65	6.25(2.558, 15.26), 0.001	2.976(0.612, 14.46)	0.176
	Total	37	283	320			
Address of health facility	Urban	19	62	81	3.76(1.86, 7.6), 0.001	1.687(0.659, 4.318)	0.275
	Rural	18	221	239	Reference		
	Total	37	283	320			

**strongly significant

## Discussion

This study investigated that more than three fourths of respondents had good knowledge towards the disease. On the contrary, in Rwanda about 60% of health professionals had good knowledge [13]. This difference might be due to efforts done by ministry of health Ethiopia and Mossy Foot Association on prevention and control measures like training health workers on LMMDP increased the knowledge of the study subjects. Our finding revealed that 22% of respondents identified podoconiosis as an infectious disease. Regarding the cause, 48, 11.9 and 22.1% said that the disease is caused by soil particles, parasite and curse/evil eye, respectively.

Fifty five percent of participants correctly answered that not wearing shoes is risk factor for podoconiosis. This is finding is almost similar with study done in Rwanda in which about 61% of study subjects knew that walking barefoot was a risk factor for acquiring podoconiosis [13]. The possible explanation for this result may be similar interventions like training to health professionals on lymphoedema management, community mobilization and sensitization in podoconiosis endemic districts has been conducted by the two countries.

According to the present study, knowledge score was significantly associated with profession, service year and attitude of the study participants. BSc nurses, health officers and diploma nurses had 11, 25 and 4 times respectively more likely to have good knowledge than other professionals like midwife, laboratory and pharmacy technicians. The possible explanation would be due to the fact that other health professionals had low exposure to disease management and training in Ethiopia.

The present study found that 56% of the study subjects had favorable attitude towards podoconiosis patients. This finding was not consistent with study done in Rwanda in that 86% of respondents expressed positive attitudes towards podoconiosis [13]. Our finding is also not in line with study conducted in Wolayta zone in which 72.4% of health professionals had favorable attitude towards providing care for podoconiosis patients [14]. This difference might be due to on job trainings and other interventions given to health professionals regarding podoconiosis in the comparative areas. It could it also be due to difference in the tool used to measure favorable attitude.

In the current study, only 8 % felt that they had adequate knowledge and skill to give care and treatment for podoconiosis patients. Forty seven (14.7%) of the study subjects believed they may acquire podoconiosis if they are in contact with podoconiosis patients. On the contrary, previously conducted study reported that about 50% of health professionals were afraid of acquiring podoconiosis from patients [14]. The possible reason might be in the previous study subjects had poor awareness towards podoconiosis transmission and misconception towards its cause.

Out of total respondents, about 13% felt they were uncomfortable if they buy food or items from a shop keeper with podoconiosis. On the other hand, twenty (6.3%) of health professionals believed people will not appreciate them if they treated podoconiosis patients. These kinds of beliefs may imply health care providers ignored the value for treatment and their profession. Our findings were supported by studies done in Ethiopia and Rwanda [13–15].

In this study, only knowledge of the participants was significantly associated with attitude towards podoconiosis patients. Those health professionals who had good knowledge were 2.4 times more likely to have favorable attitude than poor knowledge. This finding agrees with study done by Bereket Yakob in 2007 [15]. It may be possible to improve attitude by improving knowledge. This should be investigated in further research.

Regarding practice of health professionals towards management of cases of podoconiosis, very few 37/320 (11.6%) health professionals ever treated podoconiosis patients. The current study showed lower practice when compared to other previously conducted studies [14]. The low practice might be related with inadequate provision of medical supplies and in-service trainings.

Our study revealed that training on LMMDP and age category was significantly associated with having treated podoconiosis patients. Health professionals who trained on LMMDP were 7.4 times more likely to treat cases than others. On the other hand, age group 45 years old above individuals were 17 times higher chance of to be treated podoconiosis patients when compared to age group 25–34 years old. This might be possibly because of the older age groups had more experience in treating cases and exposure to managing cases.

This study has the following limitations: we did not include health professionals working in private clinics. We are also unable to include, anesthesia and environmental health workers. Although physicians are our eligible study participants, we found none in the selected health facilities. There might be recall bias that influence the response questions about whether HPs have ever treated podoconiosis. The other limitation could be our findings might not be generalizable/applicable to other countries as our subjects were limited to specific health care professionals.

## Conclusion and recommendations

Despite, high percent of good knowledge of health professionals towards podoconiosis, experience of respondents on treating cases was very low. In this study knowledge score was significantly associated with profession, service year and attitude of the study participants. Only knowledge status was significantly related with attitude of participants. Those individuals who had good knowledge have 2.4 times more likely to have favorable attitude than those who had poor knowledge score. Health professionals who trained on LMMDP and age category equals or above 45 years old were significantly associated with practices of health professionals. Future research should evaluate whether in-service trainings can improve knowledge, attitude and practice for health professionals in podoconiosis endemic districts. In podoconiosis endemic districts hygiene supplies and other referencing materials should be made available for podoconiosis case management.

## Data Availability

The datasets used and/or analyzed during the current study are available from the corresponding author on reasonable request.

## Abbreviations

AOR: Adjusted Odds Ratio
CI: Confidence Interval
HO: Health Officer
HP: Health Professionals
ICCC: Innovative Care for Chronic Conditions
IRB: Institute Research Board
LMMDP: Lymphoedema Morbidity Management and Disability Prevention
MFTPA: Mossy Foot Treatment and Prevention Association
NTD: Neglected Tropical Disease
OR: Odds Ratio
SPSS: Statistical Package for Social Sciences
VIF: Variance Inflation Factor
WHO: World Health Organization
